# Effect of Oat Flakes on Glycemic Variability, Dyslipidemia, and Pancreatic Duodenum Homeobox-1 (PDX-1) Level Among Adolescents with Type 1 Diabetes: A Randomized Crossover Study

**DOI:** 10.3390/nu18111802

**Published:** 2026-06-03

**Authors:** Mohamed Abu El Asrar Afify, Sara Ibrahim Taha, Eman Mohamed El Kholy, Nouran Yousef Salah

**Affiliations:** 1 Pediatrics Department, Faculty of Medicine, Ain Shams University, Cairo 11522, Egypt; mohamedaboelasrar@med.asu.edu.eg; 2 Clinical Pathology Department, Faculty of Medicine, Ain Shams University, Cairo 11522, Egypt; dr_sara_ib@med.asu.edu.eg; 3 Ministry of Health and Population, Cairo 11516, Egypt; eman.elkholy@med.asu.edu.eg; 4 Academy of Scientific Research and Technology, Cairo 11694, Egypt

**Keywords:** adolescents, type 1 diabetes, high-fiber diet, glycemic variability, pancreatic duodenum homeobox-1

## Abstract

**Aims:** Murine studies show a promising effect of high-fiber *β-glucan* on glycemic control and serum lipids. In addition, *β-glucan* has recently been found to have strong antioxidant and immunomodulatory effects. Oat flakes are a natural source of *β-glucan*. However, the effects of oat flakes on glycemic variability, dyslipidemia, and pancreatic duodenum homeobox-1 (PDX-1) levels in type 1 diabetes (T1D) remain unclear. Hence, this study assessed the effect of oat flakes on glycemic variability, dyslipidemia, and PDX-1 among adolescents with T1D. **Materials and Methods:** Sixty adolescents with T1D were divided into 2 equally matched groups. Group A received oat flakes *β-glucan* 6 g per day for 3 months in addition to an ordinary diet and insulin regimen. Group B received an ordinary diet and insulin regimen. This was followed by crossing over both arms for another 3 months after a two-week washout period. All participants underwent auxological assessment, continuous glucose monitoring (CGM), hemoglobin A1c (HbA1c), fasting lipids, and PDX-1 measurements at baseline, 3 months, and 6 months. **Results:** Oat flakes consumption resulted in a significant decrease in the coefficient of variation, HbA1c, serum cholesterol, triglycerides, and LDL-C levels (*p* < 0.001), with a significant increase in TIR, HDL-C, and PDX-1 levels (*p* < 0.001). However, all these effects waned after the stoppage of the oat flakes, except for HDL-C. **Conclusions:** Oat flakes have a favorable outcome on glycemic metrics, lipid profile, and PDX-1 in adolescents with T1D.

## 1. Introduction

Attaining optimal glycemic control is a cornerstone in type 1 diabetes (T1D) management, aiming at enhancing the quality of life and minimizing short- and long-term diabetes complications. This is especially important in adolescents who tend to have poor glycemic control. Postprandial glucose excursions and glucose variability are substantial challenges to children and adolescents with T1D, particularly those on a basal-bolus regimen [1]. Hence, improving the glycemic control in this vulnerable population is very crucial and challenging.

Glycemic variability and hyperlipidemia represent dual threats to pancreatic β-cells. The combination of the two, glucolipotoxicity, is particularly harmful. Many in vitro studies have shown that high concentrations of free fatty acids (FFAs) have a deleterious effect on pancreatic β-cells; in addition, in vivo infusion of FFA in humans and rodents has been found to suppress insulin secretion [2].

Pancreatic duodenum homeobox-1 (PDX-1) is a master regulator in pancreatic organogenesis, maturation, and preservation of β-cells [3]. Studies have shown increased expression of PDX-1 in type 2 diabetes (T2D), allowing its use to evaluate residual pancreatic function [4]. Moreover, PDX-1 has been linked to T1D pathogenesis and considered a marker of pancreatic β-cell reserve, with PDX-1 autoantibodies detected in individuals with T1D [3]. In addition, the potential role of PDX-1 in reversing diabetes is emerging [5].

Nutritional therapy represents a cornerstone in diabetes management. However, healthy dietary habits are not easily adopted in adolescents with T1D [6]. Current guidelines advise people with T1D to consume non-starch-containing vegetables, minimize added sugars and refined grains, and choose whole foods over highly processed foods [7], but these recommendations are largely based on the adult population and underscore the different nutritional needs of growing adolescents.

Oat flakes are rich in the viscous soluble dietary fiber *β-glucan*. *β-glucan* has several potentially beneficial physiological effects, which include reducing both postprandial glycemic responses (PPGRs) and serum cholesterol. The ability of *β-glucan* to reduce PPGR was established by the European Food Safety Authority [8]. In addition, *β-glucan* was shown recently to have a strong antioxidant and immunomodulatory effect [9]. Thus, *β-glucan* could be considered to support T1D patients in daily glycemic management. However, results from studies assessing the effect of dietary fiber intake on glycemic metrics in people with T1D are contradictory [10,11,12], and the long-term effect of oat flakes on glycemic metrics and lipid profile is still obscure. Hence, this study aimed to investigate the long-term effect of oat flakes use on glycemic metrics and lipid profile among adolescents with T1D.

## 2. Methodology

### 2.1. Study Population

A prospective open-label randomized controlled double-armed crossover study was performed at the Pediatric and Adolescent Diabetes Unit (PADU), Children’s Hospital, Ain Shams University, over one year (June 2024 to June 2025). Sixty adolescents with T1D, defined according to the International Society for Pediatric and Adolescent Diabetes (ISPAD) 2022 criteria [13], aged 12–18 years, were included. Those with coexisting autoimmune diseases affecting glycemic control (e.g., celiac, autoimmune thyroiditis), diseases that require dietary restriction (e.g., celiac disease, food allergy, eating disorders), chronic diabetes complications (e.g., gastroparesis), and other types of diabetes, e.g., type 2 diabetes mellitus (T2D), were excluded.

PASS 15 program was used for sample size calculation, reviewing results from Bozbulut et al. and assuming an effect size difference of 0.8 between the two groups regarding HbA1c % [14]. After a 10% adjustment for dropout rate, the program achieves 80% power to detect differences among the means versus the alternative of equal means using an F test with a 0.05 significance level.

### 2.2. Ethical Considerations

Approval was taken from the Institutional Review Board (IRB) and the ethical committee of the Faculty of Medicine, Ain Shams University, with the approval number FMASU MS 362/2024. Written informed consent and assent were obtained from the patients and their legal guardians before enrollment after a thorough explanation of the study design and expected outcomes using neutral language by the same investigator to all participants. This trial was registered at ClinicalTrials.gov (NCT07593105).

### 2.3. Randomization

A hundred adolescents with T1D were screened for eligibility; 18 patients did not meet the inclusion criteria, 8 patients declined to participate, 14 patients were excluded, and 60 participants were enrolled (Figure S1).

Eligible participants were randomly assigned in a 1:1 manner based on a computer-generated randomization sequence into two equally matched groups (30 participants per group) to receive oat flakes in addition to their ordinary diet, either in the first three months or the following three months, after the 2-week washout period. The study was open-label.

### 2.4. Study Procedures

In this open-label, crossover study, participants were randomly assigned to two equally matched groups. In the first three months, group A received 6 g of oat flakes *β-glucan* daily in addition to their ordinary diet, while group B received their ordinary diet alone. This was followed by crossing over both arms for another 3 months after a two-week washout period between the interventions.

*β-glucan* was obtained from a natural oat product called Premium Oat Flakes (Right Nutrition, Alexandria, Egypt, Western Manufacturing and International Trade Company, Batch No. 0001). The dose was divided equally into 2 g per breakfast, lunch, and dinner. Two grams of *β-glucan* are contained in 45 g of premium oat flakes.

All participants were educated to follow an ordinary diet (dividing the total daily energy intake into 40–50% carbohydrate, 30–40% fat with no more than 10% saturated fat and trans fatty acids, and 15–25% proteins) according to the ISPAD 2022 guidelines [15].

Forty-four adolescents were on a basal-bolus insulin regimen that included subcutaneous insulin degludec (Tresiba^®^; Novo Nordisk, Copenhagen, Denmark) as the basal insulin and insulin aspart (NovoRapid^®^, Novo Nordisk, Copenhagen, Denmark) as the mealtime insulin, and sixteen were on a Medtronic 720G pump using insulin aspart (NovoRapid^®^, Novo Nordisk, Copenhagen, Denmark).

The enrolled adolescents were subjected to the following at baseline, 3 months, and at the study endpoint:

Detailed clinical assessment laying stress on age, diabetes duration, insulin dose and regimen, and anthropometric measures with calculation of the standard deviation score (SDS) according to age and sex [16].

Continuous glucose monitoring (CGM) was recorded over two weeks using FreeStyle Libre2 plus CGM (Abbott, Chicago, IL, USA) for those on a basal-bolus regimen and Medtronic Guardian 3 CGM for those on an insulin pump (MiniMed™ 720G Insulin Pump, Northridge, CA, USA). Data was downloaded using the cloud-based LibreView^®^ system for the FreeStyle Libre 2 Plus CGM and the Medtronic CareLink™ system for the Medtronic Guardian 3 CGM. CGM metrics were assessed according to the ISPAD 2022 Clinical Practice Consensus Guidelines [17].

Laboratory assessment included fasting lipid profile, HbA1c, and PDX-1. A blood sample was withdrawn after 12 h of fasting for assessment of serum total cholesterol (TC), high-density lipoprotein cholesterol (HDL-C), and triglycerides (TG) by colorimetric enzymatic assay (Advia, Siemens Healthineers, Forchheim, Germany). Serum low-density lipoprotein cholesterol (LDL-C) was calculated using the Friedewald formula [18]. Glycated hemoglobin (HbA1c) was assessed using turbidimetric inhibition immunoassay (TINIA) via the Tina-Quant^®^ HbA1c kit supplied by Roche Diagnostics on the Roche/Hitachi Cobas^®^ c501 auto analyzer (Roche Diagnostics International Ltd., CH-6343 Rotkreuz, Switzerland) and expressed in percentage. Serum pancreatic duodenum homeobox-1 (PDX-1) level was assessed using an enzyme-linked immunosorbent assay (ELISA) kit supplied by Bioassay Technology Laboratory, Nanhu Dist., Jiaxing, China (Cat. No. E6802Hu). The kit detection range is from 7 to 1500 ng/dL, and sensitivity is 3.58 ng/dL.

### 2.5. Follow-Up

All participants were closely and clinically followed up weekly by phone calls to assess compliance to the oat flakes [19] and monitor for any signs of potential adverse effects during the study period. Participants were instructed to refrain from substantial changes in their lifestyle habits throughout the study period.

### 2.6. Study Endpoints

The primary endpoint was the change in glycemic variability (CV%) from baseline to 3 months following *β-glucan* supplementation. The secondary endpoints were the change in time in range (TIR) %, time above range (TAR) %, time below range (TBR) %, and fasting lipid profile from baseline to 3 months following oat flakes supplementation.

### 2.7. Data Management and Analysis

Data were collected, revised, coded, and entered into the Statistical Package for Social Science (IBM SPSS) for Windows, Version 27.0 (IBM Corp., Armonk, NY, USA). Quantitative data were presented as mean, standard deviations, and ranges when parametric and as median and interquartile range (IQR) when non-parametric, while qualitative variables were presented as numbers and percentages. The one-sample Kolmogorov–Smirnov test was used to test that a variable is normally distributed. Chi-square test and/or Fisher’s exact test were used for comparison between qualitative data when the expected count in any cell was found to be less than 5. An independent *t*-test was used for comparison between quantitative data with parametric distribution, while the Mann–Whitney test was used for quantitative data with non-parametric distribution. The comparison between two paired groups with quantitative data and parametric distribution was performed using the paired *t*-test, while the comparison between two paired groups with non-parametric distribution was performed using the Wilcoxon rank test. The comparison between more than two paired groups regarding quantitative data and parametric distribution was performed by using the Repeated Measures ANOVA test, while with non-parametric distribution, it was performed by using the Friedman test. Spearman correlation coefficients were used to assess the correlation between two quantitative parameters in the same group. Univariate and multivariate logistic regression analysis was used to assess the most important factors associated with glycemic variability on a *β-glucan* diet among the studied adolescents with T1D. The confidence interval was set to 95%, and the margin of error accepted was set to 5%, so the *p*-value was considered significant when <0.05.

## 3. Results

This randomized controlled double-armed crossover trial was conducted on 60 adolescents with T1D. They were 26 males (43.3%) and 34 females (56.7%) with an age range from 12 to 18 years (mean ± SD 12.94 ± 1.29 years). They were randomized into two equal groups by simple random sampling (Group A and Group B). Both groups were matched in regard to age, sex, HbA1c, insulin regimen, and total daily dose. The baseline characteristics of both groups are shown in Table 1. Group A received oat flakes for the first 3 months; then crossing over was performed with group B for another 3 months after a washout period of 2 weeks.

### 3.1. Oat Flakes and BMI

As shown in Table 2 and Table 3, oat flakes administration resulted in a significant reduction in BMI z score. In addition, BMI z score was significantly correlated with total cholesterol (*p* = 0.005), LDL-C (*p* = 0.042), and GMI% (*p* = 0.046).

### 3.2. Oat Flakes and Glycemic Metrics

**Figure 1:**
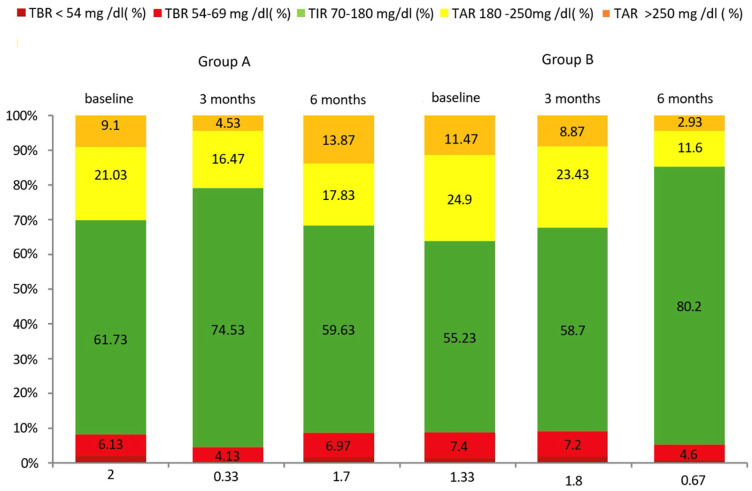
Serial follow-up of CGM metrics of groups A and B at baseline, 3, and 6 months.

As shown in Table 2 and Figure 1, a significant reduction was observed in total daily insulin dose, glycemic metrics, namely CV, HbA1c, TBR < 54 mg/dL, TBR 54–69 mg/dL, TAR 180–250 mg/dL, TAR > 250 mg/dL, and GMI during oat flakes supplementation, with a significant increase in the TIR. However, these changes were not sustained after discontinuation of the oat flakes.

### 3.3. Oat Flakes and Dyslipidemia

Regarding the lipid profile, a significant decrease in total cholesterol, triglycerides, and LDL-C was observed during oat flakes *supplementation in both groups*, *with a significant increase in HDL-C (Table 2).* These changes were not sustained after discontinuation of the oat flakes, *except for the HDL-C (Supplementary Table S1)*.

### 3.4. Oat Flakes β-Glucan and PDX-1

Interestingly, serum PDX-1 level was found to increase significantly during oat flakes supplementation (Table 2). Moreover, the CV of the studied adolescents on oat flakes supplementation was found to be significantly and independently correlated with PDX-1 on multivariate regression analysis (*p* < 0.05).

Upon comparing those on oat flakes supplementation and those on an ordinary diet, oat flakes *β-glucan* supplementation was found to cause a significant percentage change in BMI z score, insulin total daily dose, HbA1c, serum lipid profile, CGM metrics and PDX-1 compared to an ordinary diet (Table 3).

Upon comparing those on oat flakes supplementation and those on an ordinary diet, oat flakes supplementation was found to cause a significant percentage change in BMI z score, insulin total daily dose, HbA1c, serum lipid profile, and CGM metrics compared to an ordinary diet (Table 3).

Regarding the adverse effects of therapy, three patients had GIT manifestations in the form of abdominal distension and flatulence upon starting oat flakes therapy, which were mild and waned by the second week of administration, without necessitating stoppage of the oat flakes.

## 4. Discussion

Maintaining near-normal blood glycemia is essential, albeit challenging, in adolescents with T1D to prevent both short- and long-term complications. Glycemic variability has recently been recognized as the key modulator of diabetes-related complications [20].

Diabetes nutritional therapy mainly focuses on carbohydrates as the main determinant of glycemic variability, with guidelines advising for carbohydrate counting. However, limiting or eliminating carbohydrates may not be an applicable and sustainable approach, especially for adolescents; in addition, it increases the risk of hypoglycemia [21]. Owing to that, studies are approaching functional foods to reduce glycemic excursions and variability [22].

Oat flakes *β-glucan* has been shown to demonstrate several biological activities, including prebiotic, anti-diabetic, cholesterol-lowering, and immunomodulatory effects [9,10,11,12,13]. Zalecińska and colleagues reported a beneficial effect of *β-glucan* in two cases with ulcerative colitis [23]. Similarly, Oczkowski and coworkers reported that oat *β-glucan* dietary intervention has beneficial antioxidant and anti-inflammatory effects in the testes of rats with TNBS-Induced Colitis [24].

In the current study, oat flakes supplementation resulted in a significant reduction in glycemic variability, with a significant decrease in both TAR and HbA1c. This goes in line with Bozbulut and coworkers, who demonstrated a favorable outcome for *β-glucan* supplementation for 1 week on glycemic variability in 30 adolescents with T1D [14]. Similarly, a study including 16 adolescents with T1D showed that glycemic variability was negatively correlated with the total amount of dietary fiber and soluble fiber [25]. The beneficial effects of oat flakes on glycemic variability could be attributed to its high content of *β-glucan*, *which has* a glycemic index-lowering effect, through slowing down digestion and absorption of macronutrients like starch, in addition to its ability to form short-chain fatty acids secondary to anaerobic fermentation that promote PPAR-c-mediated GLUT-4 expression in the muscle fibers and adipocytes, increasing glucose uptake by myocytes and adipocytes and hence reducing blood glucose levels [26]. In contrast, a double-armed *β-glucan* placebo crossover study showed that 2 weeks of *β-glucan* did not have a significant effect on glycemic variability in adults with T1D compared to controls [12]. The lack of a significant effect in this study could be attributed to the low dose of *β-glucan used* and the lack of control for a range of factors.

It is worth mentioning that oat flakes supplementation not only reduced glycemic variability and hyperglycemia but also led to a significant reduction in TBR and hypoglycemia, which is especially important in people with T1D. This goes in line with Nader et al., who reported reduced hypoglycemia (although not statistically significant) in a cohort of 10 children with T1D on a high-fiber diet compared to a non-fiber diet [27]. Similarly, a study including 60 adults with T1D randomized to either a high-fiber or low-fiber diet for 24 weeks showed that those on the high-fiber diet had fewer hypoglycemic events [28].

The advantageous effects of oat flakes in T1D are not restricted to glycemic control. In the current study, a significant decrease in total cholesterol and LDL-C was observed during oat flakes *supplementation*, with a significant increase in HDL-C. This goes in line with a comprehensive meta-analysis that revealed that soluble fiber improves serum TG, TC, LDL-C, and Apo-B concentrations [29]. Similarly, a meta-analysis showed that the addition of *β-glucan* to diet for 2–12 weeks at a dose of ≥3 g/day could reduce LDL-C and TC in healthy individuals [30]. Another systematic review and meta-analysis showed a beneficial effect for *β-glucan* supplementation on cardiovascular disease risk markers, including blood lipids, regardless of dietary background or control [31]. This highlights the important protective role of oat flakes supplementation against dyslipidemia and diabetes-related vascular complications. Possible explanations for the beneficial effect of oat flakes include its high content of *β-glucan*, which has a gel-forming physical property that modulates host bile acid and cholesterol metabolism, potentially removing intestinal cholesterol for excretion. In addition, *β-glucan* has been shown to modulate the gut microbiota, exerting an apparent “prebiotic” effect, particularly on those bacterial species that influence host bile acid metabolism and production of short-chain fatty acids, factors that are regulators of host cholesterol homeostasis [32].

Interestingly, a continued upward trend in HDL concentration was observed even after the discontinuation of oat flakes supplementation in group A. Robert et al. reported a similar finding in a study assessing the effect of B-glucan on serum lipids in men with obesity and hypercholesterolemia. They found that HDL cholesterol concentrations did not increase immediately with consumption of *β-glucan*; rather, it took 12 weeks, which was 4 weeks after the cessation of the *β-glucan* fiber supplement [33]. This suggests that *β-glucan* might play a role in HDL cholesterol concentration increase, and indicates a beneficial carryover effect for *β-glucan* on HDL up to 12 weeks after stoppage of the *β-glucan* supplementation.

In the present study, oat flakes supplementation was associated with a significant reduction in the total daily insulin dose, reflecting improved insulin sensitivity. This goes in line with a meta-analysis by Bao et al., which reported significant reductions in fasting plasma insulin in healthy individuals, individuals with obesity, and those with T2D after long-term oat flakes *β-glucan* consumption (≥8 weeks) [34]. Similarly, a study including 8–11-year-old children revealed that high whole-grain oat intake is inversely associated with serum insulin and cardiometabolic markers [35]. In contrast, a meta-analysis by Shen and colleagues showed no significant effect of oat *β-glucan* supplementation for 3 to 8 weeks on insulin sensitivity in people with T2D [36]. The difference in the results could be attributed to the short intervention period in Shen and colleagues’ meta-analysis.

In addition to its beneficial effects on glycemia and lipid profile, oat flakes supplementation was found to decrease BMI in the studied adolescents with T1D. This is in concordance with Damsgaard and colleagues, who reported a significant association between whole-grain oat intake and lower fat mass index in healthy children [35]. Similarly, an observational study using NHANES data reported an inverse association between total whole-grain intake and BMI in 6- to 18-year-old children and adolescents [37]. In addition, two randomized controlled studies including people with T2D showed a significant reduction in BMI on dietary oat supplementation during a follow-up period of three to four weeks [38,39]. This reduction in BMI could be a potential modulator of the beneficial effects of oat flakes on glycemia, insulin resistance, and lipid profile.

In the present study, serum PDX-1, a master regulator protein in β-cell maturation and identity preservation, was found to be detectable in sera of adolescents with T1D. PDX-1 is a nuclear protein that is primarily known for its role in pancreatic development and β-cell maturation, rather than as a hormone or traditional protein biomarker that circulates at high levels in the serum of healthy individuals [3]. However, research indicates that PDX-1 can be detected in human sera of people with diabetes, people with pancreatic cancer, and healthy populations [40,41]. The presence of PDX-1 in the serum of adolescents with T1D is a novel finding that could be attributed to hormonal changes. Secretion of PDX-1 in extracellular vesicles (e.g., exosomes) under pathological stress (such as diabetes), cellular stress, and inflammation could cause leakage of nuclear proteins like PDX-1 into the extracellular space and their subsequent entry into the bloodstream. This goes in line with Zhang and colleagues, who reported that increased serum PDX-1 in early pregnancy is associated with decreased risks of gestational diabetes mellitus in a prospective study including 231 pregnant women [41]. The detection of PDX-1 in the sera of the studied adolescents with T1D is of particular interest, since these adolescents have established stage 3 T1D, with a diabetes duration ranging from 1 to 12 years. This could be interpreted by the residual pancreatic β-cells in people with longstanding T1D. Previous studies have reported longstanding β-cell function in people with T1D, which they attributed to residual pancreatic β-cells. They described intermittent rather than total eradication of the pancreatic β-cell reserve, allowing a small population to persist [42]. Similarly, a study of individuals with longstanding T1D reported clinically meaningful β-cell reserves in nearly 40% of individuals with disease durations of over 10 years, which they associated with better glucose control and reduced hypoglycemia risk [43].

Interestingly, a significant increase in serum PDX-1 level was observed during oat flakes *β-glucan* supplementation. In agreement with this, a murine study showed that *β-glucan* promotes protection from T1D through inducing mixed pro- and anti-inflammatory responses, which promote regulatory T cell (Treg) and Th17 responses, resulting in pancreatic β-cell protection [44]. Although an increase in PDX-1 levels was observed following administration of oat flakes in the current study, further and longer longitudinal studies with larger samples are required to verify the potential effect of oat flakes on PDX-1 levels and the potential patho-mechanistic role of serum PDX-1 in pancreatic reserve assessment.

## 5. Conclusions

In conclusion, oat flakes supplementation over 3 months was associated with a significant increase in TIR, HDL-C and PDX-1, along with a significant decrease in CV, TBR, triglycerides, cholesterol, and LDL-C. However, these changes were not sustained and reverted after discontinuation of the *β-glucan*, except for the HDL-C. Thus, oat flakes could be a safe adjuvant dietetic intervention to improve glycemic control and dyslipidemia among adolescents with T1D on different insulin treatment modalities.

## 6. Limitations

One limitation of the current study is the lack of standardized meals, relying on patients’ self-reported intake and the standardization of the dose of *oat flakes*. Another limitation is the use of a whole food source of *β-glucan*, as the quantity of *oat flakes* can affect satiety and other factors related to glycemic control. Moreover, the study’s follow-up duration was only 3 months per intervention. Hence, longer studies under real-life situations are warranted to confirm the best *β-glucan* dose for glycemic control in adolescents with T1D.

## Figures and Tables

**Table 1:** Baseline clinico-laboratory characteristics of the studied adolescents with T1D.

		Group A	Group B	Test Value	*p*-Value
		No. = 30	No. = 30		
Sex	Male	12 (40%)	14 (46.7%)	0.271 *	0.602
	Female	18 (60%)	16 (53.3%)		
Age (years)	Mean ± SD	13.07 ± 1.51	12.8 ± 1.06	0.792 •	0.432
	Range	12–18	12–16		
Diabetes duration (years)	Median (IQR)	3 (1.6–8)	4 (2–5)	−0.149 ≠	0.882
	Range	1–12	1.3–11		
Insulin regimen	Basal bolus regimen	20 (66.7%)	24 (80%)	1.364 *	0.243
	Insulin pump	10 (33.3%)	6 (20%)		
Total daily dose (unit/kg/day)	Mean ± SD	0.98 ± 0.24	1.09 ± 0.25	−1.839 •	0.071
	Range	0.63–1.58	0.75–1.68		
Family history of T1D	No	2 (6.7%)	4 (13.3%)	0.741 *	0.389
	Yes	28 (93.3%)	26 (86.7%)		
Weight z score	Median (IQR)	0.1 (−0.15–0.27)	−0.03 (−0.39–0.11)	−1.302 ≠	0.193
	Range	−1.58–1.11	−1.05–0.95		
Height z score	Median (IQR)	−0.34 (−0.88–0.13)	−0.29 (−0.71–−0.09)	−0.414 ≠	0.679
	Range	−2.51–0.65	−3.2–0.13		
BMI z score	Median (IQR)	0.38 (−0.04–0.62)	0.20 (−0.02–0.57)	−1.302 ≠	0.193
	Range	−0.31–1.35	−0.63–1.42		
HbA1c (%)	Mean ± SD	7.86 ± 1.51	8.82 ± 2.58	−1.759 •	0.084
	Range	6.2–11	5.8–15		
Triglycerides (mg/dL)	Mean ± SD	98.6 ± 36.35	90.53 ± 26.74	0.979 •	0.332
	Range	58–202	58–134		
Cholesterol (mg/dL)	Mean ± SD	180.07 ± 39.45	164.33 ± 19.67	1.955 •	0.055
	Range	123–248	125–186		
LDL-C (mg/dL)	Mean ± SD	102.0 ± 36.68	91.6 ± 22.45	1.325 •	0.191
	Range	50–178	62–139		
HDL-C (mg/dL)	Mean ± SD	60.8 ± 15.82	58.47 ± 9.94	0.684 •	0.497
	Range	33–92	38–70		
PDX-1 (ng/dL)	Mean ± SD	135.94 ± 65.49	111.62 ± 47.59	1.645 •	0.105
	Range	50.76–286.2	47.87–187.1		

T1D: type 1 diabetes; BMI: body mass index; HbA1c: glycated hemoglobin; LDL-C: low-density lipoprotein cholesterol; HDL-C: high-density lipoprotein cholesterol; PDX-1: pancreatic duodenum homeobox-1. *: Chi-square test; •: independent *t*-test; ≠: Mann–Whitney test;

**Table 2:** Comparison between baseline and post-treatment data on *β-glucan* and ordinary diet.

	B-Glucan Group (*n* = 60)					Ordinary Diet Group (*n* = 60)			
	Baseline		Post-Treatment	Test Value	*p*-Value	Baseline	Post-Treatment	Test Value	*p*-Value
**Weight z score**	Median (IQR)	0.1 (−0.15–0.27)	0.08 (−0.18–0.23)	−3.728 ≠	**<0.001**	0.1 (−0.15–0.27)	−0.03 (−0.29–0.11)	−0.862 ≠	0.389
	Range	−1.58–1.11	−1.82–0.99			−1.58–1.11	−1.05–0.95		
**Height z score**	Median (IQR)	−0.34 (−0.88–0.13)	−0.34 (−0.88–0.13)	−1.342 ≠	0.180	−0.34 (−0.88–0.13)	−0.25 (−0.68–−0.09)	−2.375 ≠	**0.018**
	Range	−2.51–0.65	−2.51–0.65			−2.51–0.65	−2.84–0.13		
**BMI z score**	Median (IQR)	0.38 (−0.04–0.62)	0.31 (−0.04–0.48)	−2.936 ≠	**0.003**	0.38 (−0.04–0.62)	0.2 (−0.02–0.57)	−0.921 ≠	0.357
	Range	−0.31–1.35	−0.31–1.11			−0.31–1.35	−0.83–1.42		
**Total daily dose** **(U/kg/day)**	Mean ± SD	0.98 ± 0.24	0.73 ± 0.27	7.726 •	**<0.001**	0.98 ± 0.24	1.07 ± 0.26	1.290 •	0.207
	Range	0.63–1.58	0.22–1.28			0.63–1.58	0.55–1.67		
**HbA1c (%)**	Mean ± SD	7.89 ± 1.56	6.50 ± 0.76	10.389 •	**<0.001**	7.89 ± 1.56	7.62 ± 1.39	0.270 •	0.788
	Range	5.6–12	5.2–8.3			5.6–12	5.6–12		
**Triglycerides (mg/dL)**	Mean ± SD	94.50 ± 30.22	87.70 ± 27.11	4.285 •	**<0.001**	94.50 ± 30.22	93.20 ± 26.44	−2.402 •	**0.019**
	Range	58–202	54–175			58–202	52–165		
**Cholesterol (mg/dL)**	Mean ± SD	149.20 ± 35.36	134.07 ± 26.20	9.045 •	**<0.001**	149.20 ± 35.36	162.73 ± 18.61	−3.862 •	**<0.001**
	Range	111–236	95–209			111–236	128–201		
**LDL-C (mg/dL)**	Mean ± SD	70.33 ± 35.76	54.83 ± 25.48	9.049 •	**<0.001**	70.33 ± 35.76	88.07 ± 22.53	−5.144 •	**<0.001**
	Range	25–168	21–116			25–168	48–143		
**HDL-C (mg/dL)**	Mean ± SD	60.87 ± 12.25	65.27 ± 7.24	−4.357 •	**<0.001**	60.87 ± 12.25	61.03 ± 9.09	−0.325 •	0.747
	Range	33–92	49–84			33–92	40–82		
**PDX1 (ng/dL)**	Mean ± SD	164.20 ± 43.69	1016.54 ± 401.50	−16.369 •	**<0.001**	164.20 ± 43.69	183.31 ± 77.70	−0.465 •	0.644
	Range	69.8–286.2	316.1–1474			69.8–286.2	57.3–371.8		
**TIR (%)**	Mean ± SD	60.22 ± 13.90	77.37 ± 11.30	−12.936 •	**<0.001**	60.22 ± 13.90	59.17 ± 10.41	2.903 •	**0.005**
	Range	20–90	44–100			20–90	39–81		
**TBR < 54 mg/dL (%)**	Mean ± SD	1.42 ± 2.08	0.50 ± 0.79	3.328 •	**0.002**	1.42 ± 2.08	1.75 ± 1.74	−3.418 •	**0.001**
	Range	0–9	0–3			0–9	0–7		
**TBR 54–69 mg/dL (%)**	Mean ± SD	6.80 ± 5.80	4.37 ± 3.56	3.326 •	**0.002**	6.80 ± 5.80	7.08 ± 5.32	−1.640 •	0.106
	Range	0–28	0–15			0–28	0–27		
**TAR 180–250 mg/dL (%)**	Mean ± SD	22.47 ± 12.76	14.03 ± 7.69	5.440 •	**<0.001**	22.47 ± 12.76	20.63 ± 9.60	0.043 •	0.966
	Range	0–80	0–29			0–80	4–37		
**TAR > 250 mg/dL (%)**	Mean ± SD	9.10 ± 6.21	3.73 ± 4.33	5.673 •	**<0.001**	9.10 ± 6.21	11.37 ± 7.30	−2.533 •	**0.014**
	Range	0–25	0–22			0–25	0–35		
**GMI %**	Mean ± SD	7.00 ± 0.73	6.63 ± 0.64	4.822 •	**<0.001**	7.00 ± 0.73	7.17 ± 0.81	−2.723 •	**0.008**
	Range	5.6–8.1	5.3–7.7			5.6–8.1	5.6–8.8		
**CV %**	Mean ± SD	38.61 ± 7.61	32.72 ± 7.41	5.725 •	**<0.001**	38.61 ± 7.61	39.67 ± 6.47	−3.167 •	**0.002**
	Range	18.4–48.2	17.9–46.1			18.4–48.2	26.3–55.3		

BMI: body mass index; HbA1c: glycated hemoglobinLDL-C: low-density lipoprotein cholesterol; HDL-C: high-density lipoprotein cholesterol; PDX-1: pancreatic duodenum homeobox-1; TIR: time in range; TBR: time below range; TAR: time above range; GMI: glucose management indicator; CV: coefficient of variation. Bold: *p* < 0.05: Significant (S); •: paired *t*-test; ≠: Wilcoxon rank test.

**Table 3:** Comparison between the *β-glucan* and ordinary diet groups regarding the percentage of change of the studied parameters.

Percentage Change		B-Glucan Group (*n* = 60)	Ordinary Diet Group (*n* = 60)	Test Value	*p*-Value
**Weight z score**	Median (IQR)	0 (0–13.33)	0 (0–0)	−1.651	0.099
	Range	−66.67–44.44	−34.48–22.22		
**Height z score**	Median (IQR)	0 (0–0)	0 (0–0)	−1.785	0.074
	Range	−39.84–0	−42.25–0		
**BMI z score**	Median (IQR)	0 (−26.67–0)	0 (0–0)	−3.578	**<0.001**
	Range	−61.45–0	−3.33–38.46		
**Total daily dose** **(U/kg/day)**	Median (IQR)	−20.55 (−38–−14.42)	0 (0–0)	−5.620	**<0.001**
	Range	−76.85–14.93	−34.88–18		
**HbA1c (%)**	Median (IQR)	−15.58 (−20–−10.3)	−1.87 (−8.54–10.73)	−6.919	**<0.001**
	Range	−40–−4.29	−28.57–50		
**Triglycerides(mg/dL)**	Median (IQR)	−4.35 (−8.96–−3.54)	5.07 (−4.08–11.48)	−4.756	**<0.001**
	Range	−46.55–19.4	−13.59–93.55		
**Cholesterol (mg/dL)**	Median (IQR)	−7.69 (−12.5–−4.91)	1.38 (−3.4–14.29)	−8.442	**<0.001**
	Range	−26.6–3.6	−6.4–51.58		
**LDL-C (mg/dL)**	Median (IQR)	−19.47 (−26.19–−13.8)	5.75 (1.23–17.74)	−8.971	**<0.001**
	Range	−55.13–2.44	−19.83–61.29		
**HDL-C (mg/dL)**	Median (IQR)	9.03 (4.35–13.56)	6.09 (−4.29–14.93)	−1.312	0.189
	Range	−21.18–81.82	−17.74–30.95		
**PDX1 (ng/dL)**	Median (IQR)	294.76 (203.83–395.24)	−39.22 (−53.1–−6.66)	−8.960	**<0.001**
	Range	48.87–1310.61	−79.12–268.76		
**TIR(%)**	Median (IQR)	27.66 (12.94–44.9)	−7.18 (−20–8.5)	−6.894	**<0.001**
	Range	−20–120	−48.68–51.72		
**TBR < 54 mg/dL (%)**	Median (IQR)	−100 (−100–−71.43)	−10 (−100–50)	−3.136	**0.002**
	Range	−100–50	−100–300		
**TBR 54–69 mg/dL (%)**	Median (IQR)	−43.75 (−71.43–0)	10.56 (−16.67–150)	−4.772	**<0.001**
	Range	−100–150	−100–1300		
**TAR 180–250 mg/dL (%)**	Median (IQR)	−33.33 (−55.17–3.57)	−10.26 (−33.33–16.67)	−2.985	**0.003**
	Range	−100–300	−80.77–1300		
**TAR > 250 mg/dL (%)**	Median (IQR)	−71.43 (−100–−20)	0 (−33.33–114.29)	−5.473	**<0.001**
	Range	−100–150	−100–800		
**GMI %**	Median (IQR)	−2.8 (−9.29–0)	2.01 (−1.75–8.84)	−4.836	**<0.001**
	Range	−31.25–11.76	−16.78–49.15		
**CV %**	Median (IQR)	−14.29 (−30.91–−3.43)	3.41 (−4.79–27)	−5.774	**<0.001**
	Range	−52.89–94.57	−26.54–152.51		

BMI: body mass index; HbA1c: glycated hemoglobin; LDL-C: low-density lipoprotein cholesterol; HDL-C: high-density lipoprotein cholesterol; PDX-1: pancreatic duodenum homeobox-1; TIR: time in range; TBR: time below range; TAR: time above range; GMI: glucose management indicator; CV: coefficient of variation. Bold: *p* < 0.05: Significant.

## Data Availability

Data will be available from the corresponding author upon reasonable request.

## Supplementary Materials

The following supporting information can be downloaded at: https://www.mdpi.com/article/10.3390/nu18111802/s1. Figure S1: CONSORT flow diagram for the enrolled adolescents with T1D; Table S1: Serial follow-up of group A and group B regarding various clinico-laboratory parameters at baseline, 3 and 6 months.

## References

[1] Cherubini V., Marino M., Marigliano M., Maffeis C., Zanfardino A., Rabbone I., Giorda S., Schiaffini R., Lorubbio A., Rollato S. (2021). Rethinking Carbohydrate Intake and Time in Range in Children and Adolescents with Type 1 Diabetes. Nutrients.

[2] De Wit D.F., Snethlage C.M.F., Rampanelli E., Maasen K., Walpot N., Van Raalte D.H., Nieuwdorp M., Soeters M.R., Hanssen N.M.J. (2024). Higher fiber and lower carbohydrate intake are associated with favorable CGM metrics in a cross-sectional cohort of 470 individuals with type 1 diabetes. Diabetologia.

[3] Ebrahim N., Shakirova K., Dashinimaev E. (2022). PDX1 is the cornerstone of pancreatic β-cell functions and identity. Front. Mol. Biosci..

[4] Moore A.G.S., Okoduwa S.I.R. (2026). Modulation of PDX1 gene expression and glycemic control by *Citrullus lanatus* in experimental type 2 diabetes. Sci. Rep..

[5] Zhang Y., Fang X., Wei J., Miao R., Wu H., Ma K., Tian J. (2022). PDX-1: A Promising Therapeutic Target to Reverse Diabetes. Biomolecules.

[6] Evert A.B., Dennison M., Gardner C.D., Garvey W.T., Lau K.H.K., MacLeod J., Mitri J., Pereira R.F., Rawlings K., Robinson S. (2019). Nutrition therapy for adults with diabetes or prediabetes: A consensus report. Diabetes Care.

[7] Johnson J., Franklin V.L., Shepherd A., Chau G., Keen K., Lennon S., Leveridge M., Maclean K., Nicol J., Phillipson V. (2025). Glucose variability and postprandial hyperglycemia after breakfast in children and young people with type 1 diabetes. Pediatr. Diabetes.

[8] Maffeis C., Tomasselli F., Tommasi M., Bresadola I., Trandev T., Fornari E., Marigliano M., Morandi A., Olivieri F., Piona C. (2020). Nutrition habits of children and adolescents with type 1 diabetes changed in a 10-year span. Pediatr. Diabetes.

[9] Zhong X., Wang G., Li F., Fang S., Zhou S., Ishiwata A., Tonevitsky A.G., Shkurnikov M., Cai H., Ding F. (2023). Immunomodulatory effect and biological significance of β-Glucans. Pharmaceutics.

[10] Weir G.C. (2019). Glucolipotoxicity, β-cells, and diabetes: The emperor has no clothes. Diabetes.

[11] Zurbau A., Noronha J.C., Khan T.A., Sievenpiper J.L., Wolever T.M.S. (2021). The effect of oat β-glucan on postprandial blood glucose and insulin responses: A systematic review and meta-analysis. Eur. J. Clin. Nutr..

[12] Frid A., Tura A., Pacini G., Ridderstråle M. (2017). Effect of Oral Pre-Meal Administration of Betaglucans on Glycaemic Control and Variability in Subjects with Type 1 Diabetes. Nutrients.

[13] Libman I., Haynes A., Lyons S., Pradeep P., Rwagasor E., Tung J.Y., Jefferies C.A., Oram R.A., Dabelea D., Craig M.E. (2022). ISPAD Clinical Practice Consensus Guidelines 2022: Definition, epidemiology, and classification of diabetes in children and adolescents. Pediatr. Diabetes.

[14] Bozbulut R., Şanlıer N., Döğer E., Bideci A., Çamurdan O., Cinaz P. (2020). The effect of beta-glucan supplementation on glycemic control and variability in adolescents with type 1 diabetes mellitus. Diabetes Res. Clin. Pract..

[15] Annan S.F., Higgins L.A., Jelleryd E., Hannon T., Rose S., Salis S., Baptista J., Chinchilla P., Marcovecchio M.L. (2022). ISPAD Clinical Practice Consensus Guidelines 2022: Nutritional management in children and adolescents with diabetes. Pediatr. Diabetes.

[16] Shafie A.M.E., El-Gendy F.M., Allahony D.M., Omar Z.A., Samir M.A., El-Bazzar A.N., El-Fattah M.A.A., Monsef A.A.A., Kairallah A.M., Raafet H.M. (2020). Establishment of Z-score reference of growth parameters for Egyptian school children and adolescents aged from 5 to 19 years: A cross-sectional study. Front. Pediatr..

[17] De Bock M., Codner E., Craig M.E., Huynh T., Maahs D.M., Mahmud F.H., Marcovecchio L., DiMeglio L.A. (2022). ISPAD Clinical Practice Consensus Guidelines 2022: Glycemic targets and glucose monitoring for children, adolescents, and young people with diabetes. Pediatr. Diabetes.

[18] Friedewald W.T., Levy R.I., Fredrickson D.S. (1972). Estimation of the concentration of low-density lipoprotein cholesterol in plasma, without use of the preparative ultracentrifuge. Clin. Chem..

[19] Cramer J.A., Roy A., Burrell A., Fairchild C.J., Fuldeore M.J., Ollendorf D.A., Wong P.K. (2007). Medication Compliance and Persistence: Terminology and Definitions. Value Health.

[20] Ajjan R.A. (2024). The clinical importance of measuring glycemic variability: Utilizing new metrics to optimize glycemic control. Diabetes Obes. Metab..

[21] Scott S.N., Anderson L., Morton J.P., Wagenmakers A.J.M., Riddell M.C. (2019). Carbohydrate restriction in type 1 diabetes: A realistic therapy for improved glycemic control and athletic performance?. Nutrients.

[22] De Torres-Sánchez A., Ampudia-Blasco F.J., Murillo S., Bellido V., Amor A.J., Mezquita-Raya P. (2025). Proposed Practical Guidelines to Improve Glycemic Management by Reducing Glycemic Variability in People with Type 1 Diabetes Mellitus. Diabetes Ther..

[23] Zalecińska A., Harasym J., Dziendzikowska K., Sikorska K., Gromadzka-Ostrowska J. (2025). Clinical Outcomes of Oat Beta-Glucan Nutritional Intervention in Ulcerative Colitis: Case Reports of a Female and a Male Patient. Nutrients.

[24] Oczkowski M., Dziendzikowska K., Pasternak-Winiarska A., Jarmołowicz K., Gromadzka-Ostrowska J. (2024). Oat Beta-Glucan Dietary Intervention on Antioxidant Defense Parameters, Inflammatory Response and Angiotensin Signaling in the Testes of Rats with TNBS-Induced Colitis. Nutrients.

[25] Peairs A.D., Shah A.S., Summer S., Hess M., Couch S.C. (2017). Effects of the dietary approaches to stop hypertension (DASH) diet on glucose variability in youth with Type 1 diabetes. Diabetes Manag..

[26] Fuse Y., Higa M., Miyashita N., Fujitani A., Yamashita K., Ichijo T., Aoe S., Hirose T. (2020). Effect of High β-glucan Barley on Postprandial Blood Glucose and Insulin Levels in Type 2 Diabetic Patients. Clin. Nutr. Res..

[27] Song Y.-J., Sawamura M., Ikeda K., Igawa S., Yamori Y. (2000). Soluble Dietary Fibre Improves Insulin Sensitivity by Increasing Muscle Glut-4 Content in Stroke-Prone Spontaneously Hypertensive Rats. Clin. Exp. Pharmacol. Physiol..

[28] Nader N., Weaver A., Eckert S., Lteif A. (2014). Effects of fiber supplementation on glycemic excursions and incidence of hypoglycemia in children with type 1 diabetes. Int. J. Pediatr. Endocrinol..

[29] Giacco R., Parillo M., Rivellese A.A., Lasorella G., Giacco A., D’Episcopo L., Riccardi G. (2000). Long-term dietary treatment with increased amounts of fiber-rich, low-glycemic-index natural foods improves blood glucose control and reduces the number of hypoglycemic events in type 1 diabetic patients. Diabetes Care.

[30] Ghavami A., Ziaei R., Talebi S., Barghchi H., Nattagh-Eshtivani E., Moradi S., Rahbarinejad P., Mohammadi H., Ghasemi-Tehrani H., Marx W. (2023). Soluble fiber supplementation and serum lipid profile: A systematic review and dose-response meta-analysis of randomized controlled trials. Adv. Nutr..

[31] de Morais Junior A.C., Schincaglia R.M., Viana R.B., Armet A.M., Prado C.M., Walter J., Mota J.F. (2022). The separate effects of whole oats and isolated beta-glucan on lipid profile: A systematic review and meta-analysis of randomized controlled trials. Clin. Nutr. ESPEN.

[32] Llanaj E., Dejanovic G.M., Valido E., Bano A., Gamba M., Kastrati L., Minder B., Stojic S., Voortman T., Marques-Vidal P. (2022). Effect of oat supplementation interventions on cardiovascular disease risk markers: A systematic review and meta-analysis of randomized controlled trials. Eur. J. Nutr..

[33] Nicolosi R., Bell S., Bistrian B., Greenberg I., Forse R., Blackburn G. (1999). Plasma lipid changes after supplementation with β-glucan fiber from yeast. Am. J. Clin. Nutr..

[34] Bao L., Cai X., Xu M., Li Y. (2014). Effect of oat intake on glycaemic control and insulin sensitivity: A meta-analysis of randomised controlled trials. Br. J. Nutr..

[35] Damsgaard C., Biltoft-Jensen A., Tetens I., Michaelsen K., Lind M., Astrup A., Landberg R. (2017). Whole-Grain Intake, Reflected by Dietary Records and Biomarkers, Is Inversely Associated with Circulating Insulin and Other Cardiometabolic Markers in 8- to 11-Year-Old Children. J. Nutr..

[36] Shen X.L., Zhao T., Zhou Y., Shi X., Zou Y., Zhao G. (2016). Effect of Oat β-Glucan Intake on Glycaemic Control and Insulin Sensitivity of Diabetic Patients: A Meta-Analysis of Randomized Controlled Trials. Nutrients.

[37] Albertson A.M., Reicks M., Joshi N., Gugger C.K. (2015). Whole grain consumption trends and associations with body weight measures in the United States: Results from the cross sectional National Health and Nutrition Examination Survey 2001–2012. Nutr. J..

[38] Reyna N.Y., Cano C., Bermudez V.J., Medina M.T., Souki A.J., Ambard M., Nuñez M., Ferrer M.A., Inglett G.E. (2003). Sweeteners and beta-glucans improve metabolic and anthropometrics variables in well controlled type 2 diabetic patients. Am. J. Ther..

[39] Ma X., Gu J., Zhang Z., Jing L., Xu M., Dai X., Jiang Y., Li Y., Bao L., Cai X. (2013). Effects of Avena nuda L. on metabolic control and cardiovascular disease risk among chinese patients with diabetes and meeting metabolic syndrome criteria: Secondary analysis of a randomized clinical trial. Eur. J. Clin. Nutr..

[40] Duarte-Medrano G., Lopez-Méndez I., Ramírez-Luna M.Á., Valdovinos-Andraca F., Cruz-Martínez R., Medina-Vera I., Pérez-Monter C., Téllez-Ávila F.I. (2019). Analysis of circulating blood and tissue biopsy pdx1 and msx2 gene expression in patients with pancreatic cancer: A case-control experimental study. Medicine.

[41] Zhang Q., Zhang Q.Q., Dong S.Q., Liu X., Wei J., Li K., Lu Y. (2025). PDX1 in early pregnancy is associated with decreased risks of gestational diabetes mellitus and adverse pregnancy outcomes. Front. Endocrinol..

[42] Lam C.J., Jacobson D.R., Rankin M.M., Cox A.R., Kushner J.A. (2017). β Cells Persist in T1D Pancreata Without Evidence of Ongoing β-Cell Turnover or Neogenesis. J. Clin. Endocrinol. Metab..

[43] Cheng J., Yin M., Tang X., Yan X., Xie Y., He B., Li X., Zhou Z. (2021). Residual β-cell function after 10 years of autoimmune type 1 diabetes: Prevalence, possible determinants, and implications for metabolism. Ann. Transl. Med..

[44] Karumuthil-Melethil S., Gudi R., Johnson B.M., Perez N., Vasu C. (2014). Fungal β-Glucan, a Dectin-1 Ligand, Promotes Protection from Type 1 Diabetes by Inducing Regulatory Innate Immune Response. J. Immunol..

