# Supplementary material for: Effect of Oat Flakes on Glycemic Variability, Dyslipidemia, and Pancreatic Duodenum Homeobox-1 (PDX-1) Level Among Adolescents with Type 1 Diabetes: A Randomized Crossover Study
Source: Nutrients. 2026 Jun 3;18(11):1802. doi: 10.3390/nu18111802 (PMC13258507; doi:10.3390/nu18111802)
Supplement: Supplementary file 1 [file nutrients-18-01802-s001.zip › nutrients-4277842-supplementary.pdf]

Supplementary table S1: Serial follow-up of group A and group B regarding various clinico-laboratory parameters at baseline, 3, and 6 months.

		Group A			Test value	P-value	Group B			Test value	P-value
		Baseline	At 3 month	At 6 month			Baseline	At 3 month	At 6 month		
**HbA1c (%)**	Mean ± SD	7.86 ± 1.51	6.52 ± 0.77	7.33 ± 1.05	23.796•	**<0.001**	8.82 ± 2.58	7.92 ± 1.62	6.47 ± 0.75	36.691•	**<0.001**
	Range	6.2 – 11	5.2 – 7.5	5.8 – 9.4			5.8 – 15	5.6 – 12	5.2 – 8.3		
**Triglycerides (mg/dl)**	Mean ± SD	98.6 ± 36.35	87.73 ± 31.43	96 ± 30	7.932•	**0.001**	90.53 ± 26.74	90.4 ± 22.4	88 ± 23	2.623•	0.100
	Range	58 – 202	55 – 175	52 – 165			58 – 134	64 – 128	54 – 123		
**Cholesterol (mg/dl)**	Mean ± SD	180.07 ± 39.45	146.60 ± 30.56	164.27 ± 21.1	57.317•	**<0.001**	164.33 ± 19.67	161.20 ± 15.97	142.33 ± 15.85	491.904•	**<0.001**
	Range	123 – 248	95 – 209	128 – 201			125 – 186	142 – 190	123 – 173		
**LDL-C (mg/dl)**	Mean ± SD	102.0 ± 36.68	70.53 ± 25.52	80.13 ± 19.46	29.688•	**<0.001**	91.6 ± 22.45	87.47 ± 16.99	58.67 ± 16.87	441.879•	**<0.001**
	Range	50 – 178	31 – 116	48 – 112			62 – 139	62 – 115	35 – 88		
**HDL-C (mg/dl)**	Mean ± SD	60.8 ± 15.82	64.4 ± 8.24	68.13 ± 7.65	5.398•	**0.018**	58.47 ± 9.94	60.93 ± 7.4	66.13 ± 6.1	41.152•	**<0.001**
	Range	33 – 92	49 – 84	51 – 82			38 – 70	43 – 70	53 – 77		
**PDX1 (ng/dl)**	Mean ± SD	135.94 ± 65.49	957.05 ± 476.44	237.72 ± 97.01	104.32•1	**<0.001**	111.62 ± 47.59	268.25 ± 57.15	1076.02 ± 306.02	318.456•	**<0.001**
	Range	50.76 – 286.2	316.1 – 1474	57.3 – 371.8			47.87 – 187.1	152.1 – 392.3	597.4 – 1410		
**TIR (%)**	Mean ± SD	61.73 ± 17.08	74.53 ± 13.75	59.63 ± 11.11	19.925≠	**<0.001**	55.23 ± 12.48	58.70 ± 9.84	80.20 ± 7.37	115.720≠	**<0.001**
	Range	20 – 90	44 – 98	39 – 81			29 – 75	44 – 80	65 – 100		
**TBR < 54 mg/dl (%)**	Mean ± SD	2.00 ± 3.18	0.33 ± 0.61	1.70 ± 1.42	6.646≠	**0.009**	1.33 ± 1.69	1.80 ± 2.04	0.67 ± 0.92	3.622≠	**0.034**
	Range	0 – 13	0 – 2	0 – 5			0 – 5	0 – 7	0 – 3		
**TBR 54-69 mg/dl (%)**	Mean ± SD	6.13 ± 4.39	4.13 ± 4.28	6.97 ± 3.61	4.364≠	**0.018**	7.40 ± 3.78	7.20 ± 6.68	4.60 ± 2.70	4.544≠	**0.019**
	Range	0 – 12	0 – 15	2 – 16			1 – 15	0 – 27	0 – 9		
**TAR 180 - 250 mg/dl (%)**	Mean ± SD	21.03 ± 15.01	16.47 ± 8.92	17.83 ± 7.97	2.433	**0.103**	24.90 ± 9.39	23.43 ± 10.38	11.60 ± 5.33	33.952	**<0.001**
	Range	0 – 80	0 – 29	4 – 30			10 – 40	5 – 37	0 – 21		
**TAR > 250 mg/dl (%)**	Mean ± SD	9.10 ± 6.81	4.53 ± 5.58	13.87 ± 8.23	15.952	**<0.001**	11.47 ± 7.47	8.87 ± 5.28	2.93 ± 2.39	30.702	**<0.001**
	Range	0 – 25	0 – 22	1 – 35			3 – 25	0 – 18	0 – 8		
**GMI %**	Mean ± SD	6.85 ± 0.71	6.73 ± 0.6	7.18 ± 0.89	4.099•	**0.022**	7.00 ± 0.75	7.15 ± 0.74	6.54 ± 0.68	13.225•	**<0.001**
	Range	5.7 – 8.1	5.7 – 7.7	5.7 – 8.8			5.35 – 7.85	5.6 – 8.1	5.3 – 7.7		
**CV %**	Mean ± SD	36.77 ± 9.05	32.8 ± 7.5	38.88 ± 7.41	13.107•	**<0.001**	40.01 ± 6.22	40.5 ± 5.4	32.61 ± 7.40	22.417•	**<0.001**
	Range	18.4 – 47.5	17.9 – 42.4	26.3 – 55.3			29 – 48.8	30.3 – 48.2	18.4 – 46.1		

HbA1c: Glycated hemoglobin; LDL-C: low-density lipoprotein cholesterol; HDL-C: high-density lipoprotein cholesterol; PDX-1: Pancreatic duodenum homeobox-1; TIR: time in range; TBR: time below range; TAR: time above range; GMI: glucose management indicator; CV: coefficient of variation.

p-value < 0.05: bold (significant).

•: Repeated Measures ANOVA test; ≠: Friedman test

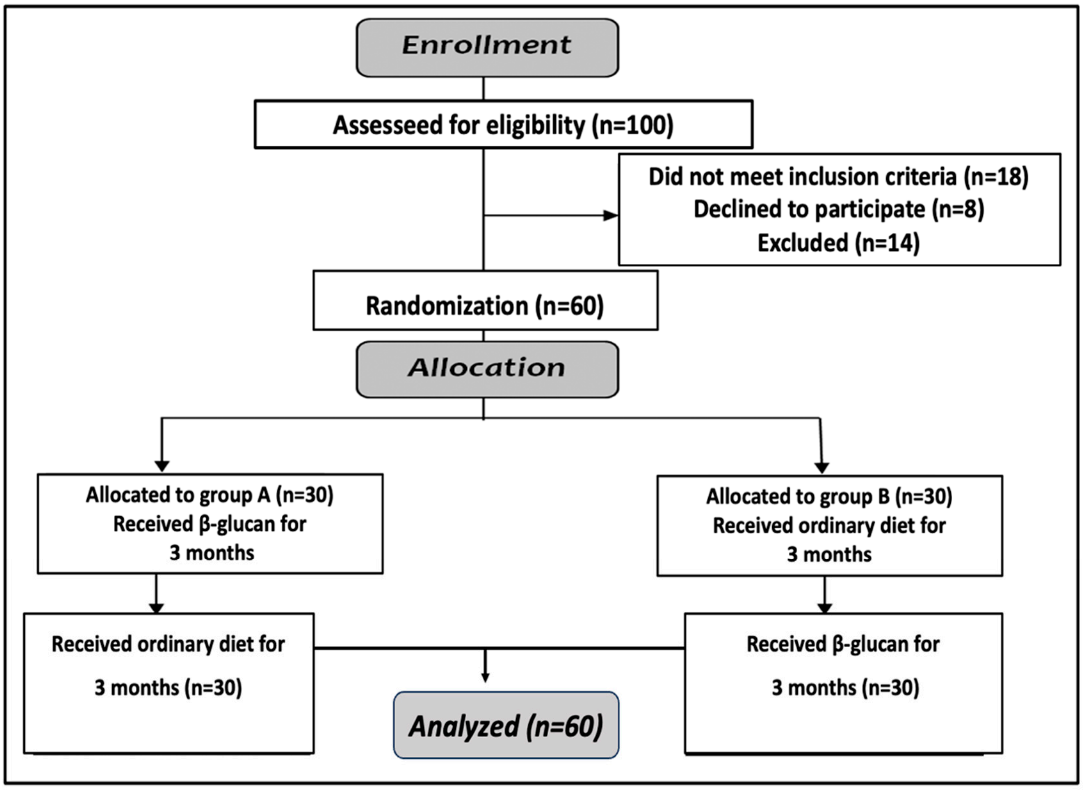

Supplementary Figure 1: CONSORT flow diagram for the enrolled adolescents with T1D
